# Cell-Permeable *Oct4* Gene Delivery Enhances Stem Cell-like Properties of Mouse Embryonic Fibroblasts

**DOI:** 10.3390/ijms22179357

**Published:** 2021-08-28

**Authors:** Da Hyeon Choi, Kyeong Eun Lee, Jiwon Park, Yoon Jeong Park, Jue-Yeon Lee, Yoon Shin Park

**Affiliations:** 1 Department of Biological Sciences and Biotechnology, School of Biological Sciences, College of Natural Sciences, Chungbuk National University, Cheongju 28644, Korea; dahyeon@cbnu.ac.kr (D.H.C.); dlruddms1223@naver.com (K.E.L.); athena_jiwon@naver.com (J.P.); 2 Department of Dental Regenerative Bioengineering and Dental Research Institute, School of Dentistry, Seoul National University, Seoul 03080, Korea; parkyj@snu.ac.kr; 3 Central Research Institute, Nano Intelligent Biomedical Engineering Corporation (NIBEC), School of Dentistry, Seoul National University, Seoul 03080, Korea; yeon0417@nibec.co.kr

**Keywords:** stemness, *Oct4*, cell-permeable peptide, direct conversion, stem cell-like properties

## Abstract

Direct conversion of one cell type into another is a trans-differentiation process. Recent advances in fibroblast research revealed that epithelial cells can give rise to fibroblasts by epithelial-mesenchymal transition. Conversely, fibroblasts can also give rise to epithelia by undergoing a mesenchymal to epithelial transition. To elicit stem cell-like properties in fibroblasts, the *Oct4* transcription factor acts as a master transcriptional regulator for reprogramming somatic cells. Notably, the production of gene complexes with cell-permeable peptides, such as low-molecular-weight protamine (LMWP), was proposed to induce reprogramming without cytotoxicity and genomic mutation. We designed a complex with non-cytotoxic LMWP to prevent the degradation of *Oct4* and revealed that the positively charged cell-permeable LMWP helped condense the size of the *Oct4*-LMWP complexes (1:5 N:P ratio). When the *Oct4*-LMWP complex was delivered into mouse embryonic fibroblasts (MEFs), stemness-related gene expression increased while fibroblast intrinsic properties decreased. We believe that the *Oct4*-LMWP complex developed in this study can be used to reprogram terminally differentiated somatic cells or convert them into stem cell-like cells without risk of cell death, improving the stemness level and stability of existing direct conversion techniques.

## 1. Introduction

Trans-differentiation processes can directly convert one cell type into another [1]. During these processes, transcription factors can induce the direct conversion or reprogramming of somatic cells into other differentiated lineages without an intermediate stage, which is important for the development of navigator cells [2,3,4,5]. Fibroblasts are abundant connective tissue cells that are biologically dynamic and morphologically heterogeneous, with diverse structures depending on their location and activity [6,7]. The main function of fibroblasts is to maintain the structural integrity of connective tissues by continuously secreting extracellular matrix precursors. Recent advances in our knowledge of the pathophysiologic features of fibroblasts revealed that while epithelial cells can give rise to fibroblasts by epithelial–mesenchymal transition, fibroblasts may also give rise to epithelia by undergoing a mesenchymal to epithelial transition [8,9,10]. 

Given their ability to differentiate into other cells, fibroblasts may provide a novel clue for in situ tissue repair and contribute to the cellular mechanisms of mesenchymal stem cell-like features in normal or pathological conditions [11,12]. Takahashi and Yamanaka [13] reported that inducible pluripotency in fibroblast cells represents a major breakthrough in cellular reprogramming. The advantage of this regenerative therapeutic technology is that the repair of damaged tissue can be generated from fibroblasts collected from a relatively easier isolation method than mesenchymal stem cell acquisition [10,14,15]. To elicit stem cell-like properties in fibroblasts, many studies have suggested that cellular functionality can be improved by regulating the tissue microenvironment [16,17,18]. Furthermore, factor-driven reprogramming of fibroblasts has been reported to directly yield other somatic cell types, such as cardiomyocytes [19], chondrocytes [20], hepatocytes [21], blood [9,22], and neural progenitors [23,24]. These reports demonstrated the ability to generate progenitors and deliver stemness factors directly without establishing pluripotency. 

Among the stemness factors, *Oct4* (also called POU5F1) is a transcription factor that functions as a master regulator during the reprogramming of somatic cells, as a transcriptional activator, and plays an essential role in the reprogramming or direct conversion of somatic cells [25]. Additionally, the expression of key hematopoietic regulators and reprogramming with remodeling factors such as Brg1, Baf155, and INO80 act as partners of *Oct4* [25]. Many strategies have been reported to transduce transcription factors, including *Oct4*, in cells by gene delivery [11,26,27]. However, the cell membrane consists of a phospholipid bilayer with embedded proteins and carries a net negative charge; therefore, biomaterials that have a positive charge are required to transport the genes through the cell membrane.

The commonly used method for reprogramming somatic cells is the introduction of transcription factors by delivering their DNA using viruses [28]. However, viral delivery has been reported to cause tumorigenicity due to unwanted genomic mutations [29]. Other methods for delivering genes includes the use of excisable transposons or non-integrating episomal vectors that can promote the non-specific insertion of DNA into the chromosome, but they have demonstrable cytotoxicity and low transfection efficiency [30]. The application of cell-permeable peptides (CPP), such as low-molecular-weight protamine (LMWP), was proposed to induce reprogramming without cytotoxicity and genomic mutation in the direct treatment into the somatic cells [31,32,33].

Herein, we propose a complex of the LMWP peptide and *Oct4* gene to induce stem cell-like properties in fibroblasts. We demonstrate that the *Oct4*-LMWP complex was activated in fibroblasts, and this activation increased stem cell-like properties in this cell system. It is anticipated that the *Oct4*-LMWP complex can be used to reprogram terminally differentiated somatic cells or give them stem cell-like properties without risk of cell death by improving stemness level and stability of existing direct conversion technologies. 

## 2. Results

### 2.1. Characterization of the Cell-Permeable Peptide, LMWP

**Figure 1:**
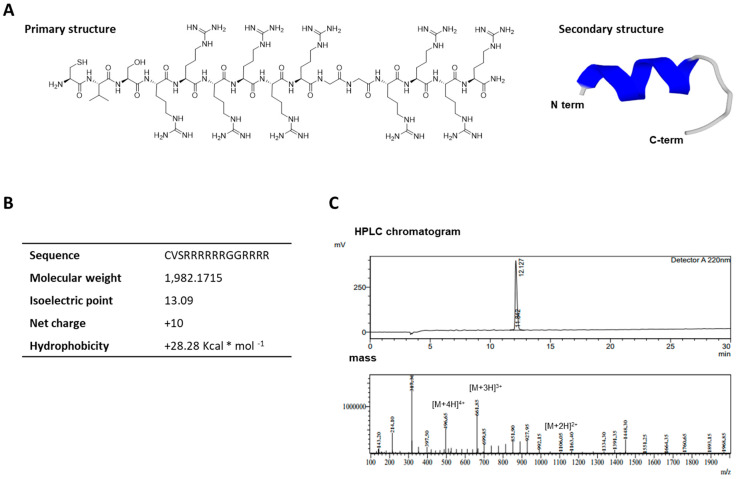
Synthesis of the LMWP cell-permeable peptide. (**A**) The primary and secondary structure of LMWP. (**B**) Sequence information of the synthesized LMWP. (**C**) HPLC chromatogram peak and mass value of LMWP.

The primary and secondary structures of the synthetic LMWP derived from human protamine are shown in Figure 1. The LMWP sequence is composed of 15 amino acids (CVSRRRRRRGGRRRR) and the N-terminus of LMWP has an α-helix structure (Figure 1A). The guanidine group makes a hole in the lipid bilayer of the cell’s membrane, which closes after LMWP internalization. Therefore, ten guanidine groups of arginine residues in LMWP can effectively induce the penetration of cells or tissues. The molecular mass of the synthesized LMWP was 1982.1715 (Figure 1B) and the purity of LMWP was 99% (Figure 1C).

### 2.2. Characterization of the Oct4-LMWP Complex

**Figure 2:**
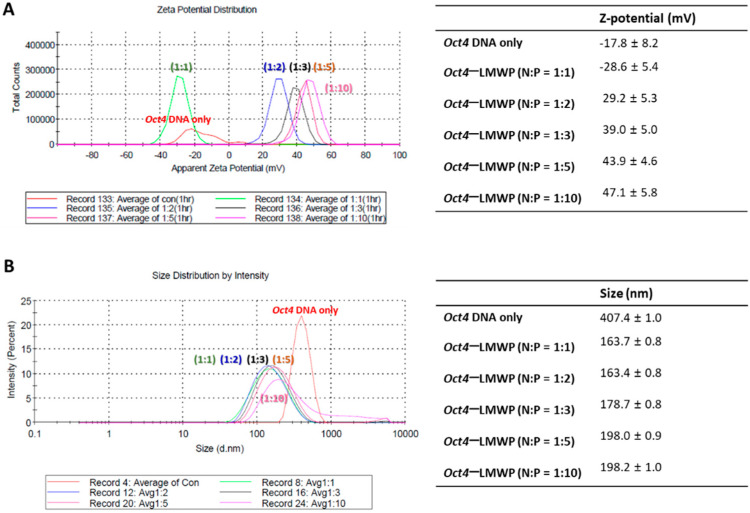
Size and surface charge distribution of the *Oct4*-LMWP complex. (**A**) Distribution of zeta potential and (**B**) size distribution of the naked *Oct4* DNA and *Oct4*-LMWP complex at different N:P molar ratios.

The *Oct4*-LMWP complexes with different molar ratios with LMWP (N:P = 1:1, 1:2, 1:3, 1:5, and 1:10) were formulated and applied for measuring their surface charge and size. The distribution of the mean zeta potential and particle size diameter of *Oct4*-LMWP complexes were determined for each of the five tested ratios (Figure 2). The apparent zeta potential distribution of *Oct4* DNA was −17.8 ± 8.2 mV (Figure 2A). The *Oct4*-LMWP complexes had zeta potential distributions of −28.6 ± 5.4 mV (N:P = 1:1), 29.2 ± 5.3 mV (N:P = 1:2), 39.0 ± 5.0 mV (N:P = 1:3), 43.9 ± 4.6 mV (N:P = 1:5), and 47.1 ± 5.8 mV (N:P = 1:10), respectively. The zeta potential of the *Oct4* DNA increased to 47.1 ± 5.8 mV when in a complex formation with LMWP. The mean particle size of the *Oct4*-LMWP complex was decreased to less than 200 nm, which was significantly smaller than that of native *Oct4* DNA (407.4 ± 1.0 nm; Figure 2B). 

### 2.3. Assessing the Stability of the Oct4-LMWP Complex

**Figure 3:**
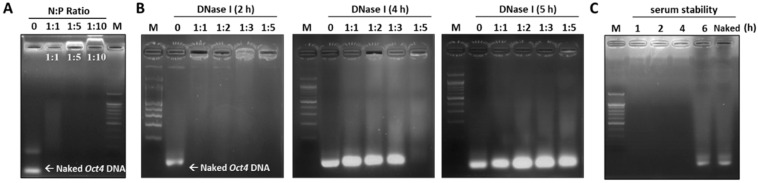
Gel retardation and serum stability assays of the *Oct4*-LMWP complex. To determine the optimum N:P molar ratio of complexes, (**A**) *Oct4* DNA and LMWP were mixed at different molar ratios and analyzed by gel retardation with 1% agarose gel electrophoresis. (**B**) The DNase I protection and (**C**) serum (50%) stability of the *Oct4*-LMWP complex (N:P = 1:5) were evaluated using an agarose gel retardation method at the indicated time points. M: size marker.

The stability of the *Oct4*-LMWP complex against enzyme and serum treatment was assessed using an agarose gel retardation assay (Figure 3). To form *Oct4*-LMWP, different ratios of CPP (1:1, 1:5, and 1:10) were added to *Oct4* DNA, while naked *Oct4* DNA was used as a control (Figure 3A). While the *Oct4*-LMWP complex (N:P = 1:10) had an upward migration pattern, the naked *Oct4* DNA rapidly migrated downward within 20 min. The *Oct4*-LMWP complexes (N:P = 1:1 and 1:5) were maintained inside the loading comb, indicating the optimum N:P molar ratio of the complex was 1:1 and 1:5 (Figure 3A). We then examined the stability of the *Oct4*-LMWP complex against nuclease degradation. DNase I (50 units) was added to different molar ratios of the *Oct4*-LMWP complex, followed by incubation of the reaction mixtures for 2, 4, or 5 h at 37 °C. *Oct4*-LMWP (N:P = 1:5) was stable for 4 h, indicating the complex was protected against nuclease digestion (Figure 3B). We next investigated the serum stability of the *Oct4*-LMWP complex (N:P = 1:5) with incubation in 50% FBS solution for 1, 2, 4, and 6 h. Naked *Oct4* DNA was used as a control and rapidly migrated downward; however, no migration of the *Oct4*-LMWP complex was observed until 4 h (Figure 3C). According to the gel retardation, DNase I, and serum stability assays, the N:P ratio of 1:5 was the optimal ratio for *Oct4*-LMWP complex formation and was stable and active for at least 4 h under the DNase I and serum-containing conditions.

### 2.4. Intracellular Uptake of the Oct4-LMWP Complex

**Figure 4:**
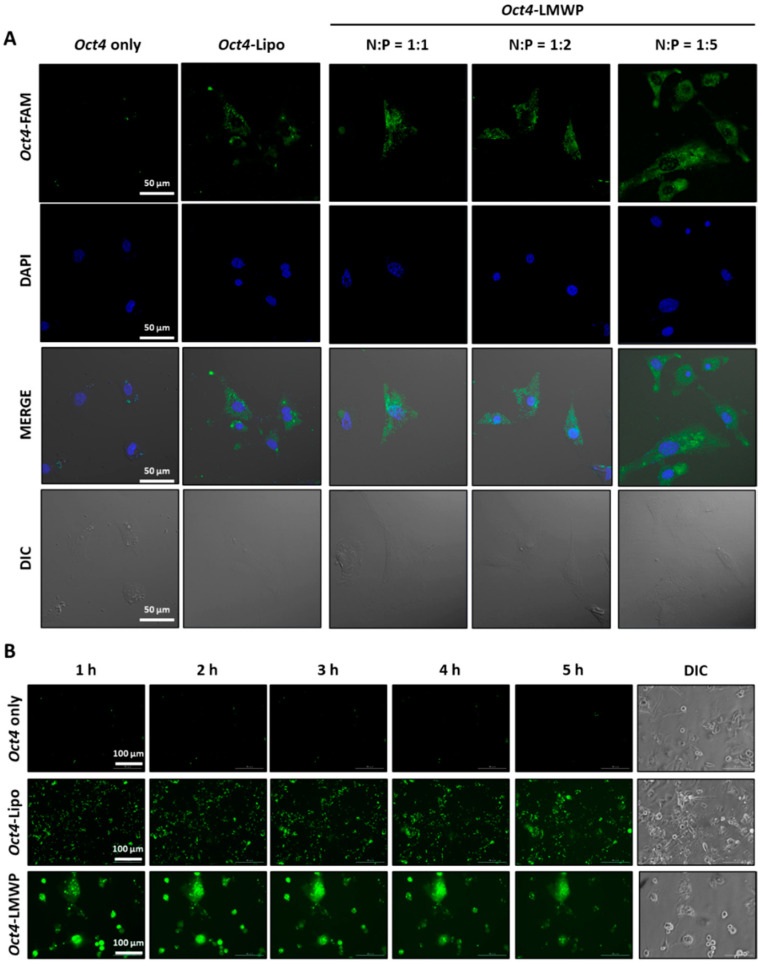
Intracellular uptake of *Oct4*-LMWP. (**A**) The cellular uptake and intracellular localization of the *Oct4*-LMWP complex were monitored at different molar ratios and analyzed using a confocal microscopy. Scale bar = 50 μm. (**B**) Real-time detection of cellular uptake of *Oct4*-LMWP complexes by MEF cells. Scale bar = 100 μm.

The cellular uptake and intracellular localization of the *Oct4*-LMWP complex were analyzed using MEF cells by confocal microscopy and real-time live imaging (Figure 4). To determine complex delivery efficacy, cellular uptake of naked *Oct4* DNA, lipofectamine-transfected *Oct4* DNA (*Oct4*-Lipo), and the *Oct4*-LMWP complex at varying N:P ratios were investigated by pre-labeling *Oct4* with the FAM fluorophore and examining their intracellular distributions. As shown in Figure 4A,B, no intercellular fluorescence was observed in the naked *Oct4* DNA samples. In cells transfected with *Oct4* DNA (*Oct4*-Lipo), fluorescence was observed in the intracellular region, while the apparent cell-permeable capacity increased in *Oct4*-LMWP-treated cells in an N:P molar-ratio-dependent manner. The complex with an N:P ratio of 1:5 showed the most apparent intracellular uptake capacity. Thus, the 1:5 *Oct4*-LMWP complex was chosen as the optimum condition for *Oct4* delivery to induce the direct conversion of MEF into MSC-like cells and was used for further experiments. The delivery efficacy of this *Oct4*-LMWP complex was monitored for 5 h. In comparison with naked *Oct4* and *Oct4*-Lipo, the *Oct4*-LMWP complex maintained its intracellular distribution for 5 h. 

### 2.5. Changes in MEF Cell Surface Markers by the Oct4-LMWP Complex Delivery

**Figure 5:**
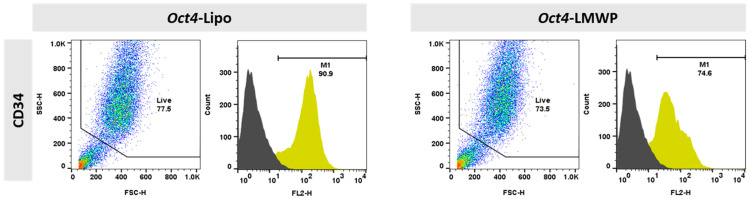
Surface marker expression in *Oct4*-treated MEF cells was characterized and compared using FACS analysis. *Oct4* was delivered by either lipofectamine (*Oct4*-lipo) or in complex with LMWP (*Oct4*-LMWP). CD34 fibroblast-specific cell surface marker was examined in each delivery system. Negative control peak is indicated as dark gray color, CD34 are indicated as yellow peaks, respectively.

The phenotypic characteristics of the *Oct4*-Lipo and *Oct4*-LMWP complexes were assessed using CD34 as a representative fibroblast surface marker (Figure 5). Using FACS, we found that 90.9% of *Oct4*-Lipo cells expressed CD34, while, in comparison, 74.6% of cells treated with *Oct4*-LMWP expressed CD34. These results indicated a decrease in fibroblast-specific characteristics when *Oct4* was delivered with LMWP. 

The CD34 expression (yellow peak) results indicated that *Oct4*-delivered MEF cells presented increased stemness, which was determined by increased CD34 surface marker expression and decreased fibroblastic characteristics. 

### 2.6. Fibroblast-Specific and Stemness Marker Gene Expression in Response to Oct4-LMWP Treatment 

**Figure 6:**
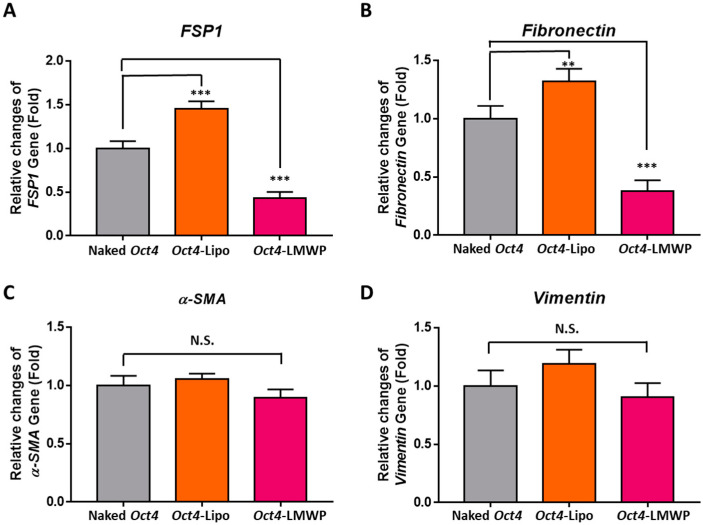
Gene expression of fibroblast-specific markers in MEF cells. Changes in the expression of fibroblast-specific marker genes (**A**) *FSP1*, (**B**) *fibronectin*, (**C**) *a-SMA*, and (**D**) *vimentin* in MEF cells with *Oct4* delivered by lipofectamine or *Oct4*-LMWP complex were confirmed by quantitative RT-PCR. The bar graph represents the relative fold changes in gene expression, which were determined by the cT value of each sample normalized to that of *β-actin*. Statistical significance between groups was analyzed using the Student’s *t*-test. The level of significance is represented as ** *p* < 0.001, *** *p* < 0.001.

The changes in fibroblast-specific marker gene expression in *Oct4*-delivered MEF cells were examined by quantitative RT-PCR analysis of *fibroblast*-*specific protein* (*FSP1*), *fibronectin*, *α-smooth muscle actin* (*α-SMA*), and *vimentin* (Figure 6A–D). With *Oct4*-LMWP treatment, the *FSP1* and *fibronectin* gene expression was significantly decreased 0.43 ± 0.07-fold and 0.37 ± 0.09-fold compared to naked *Oct4* treated MEF cells, respectively; there was no significant change in *α-SMA* and *vimentin* expression in these cells.

**Figure 7:**
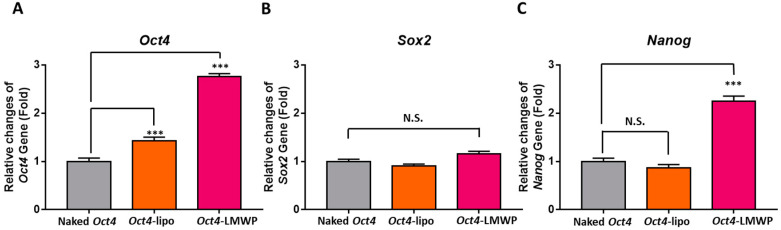
Gene expression of stemness markers in MEF cells. Changes in the expression of stemness marker genes (**A**) *Oct4*, (**B**) *Sox2*, and (**C**) *Nanog* in MEF cells treated with *Oct4*-Lipo or *Oct4*-LMWP complexes were confirmed by quantitative RT-PCR. The bar graph represents the relative fold changes in gene expression, which were determined by the cT value of each sample normalized to that of *β-actin*. Statistical significance between groups was analyzed using the Student’s *t*-test. The level of significance is represented as *** *p* < 0.001.

The change expression of stem cell-specific marker genes, including *Oct4*, *Sox2*, and *Nanog*, with *Oct4* delivery was further evaluated (Figure 7). *Oct4*-Lipo cells showed significantly increased expression that was 1.4 ± 0.1-fold compared to control MEF cells. However, there were no significant change in *Sox2* and *Nanog* gene expression. The *Oct4*-LMWP-treated group presented significantly increased expression of *Oct4* and *Nanog* genes that were 2.8 ± 0.1- and 2.3 ± 0.1-fold, respectively, compared to the control. Although there were few differences in *Sox2* gene expression among the experimental groups, *Oct4*-LMWP cells showed an increased pattern in the *Sox2* gene in naked *Oct4* treated MEFs and *Oct4*-Lipo groups. These results suggest that *Oct4*-delivered MEFs possess improved MSC-specific characteristics, indicating increased expression of stem cell marker genes. 

## 3. Discussion

The current study demonstrates the direct conversion of MEFs into MSC-like stem cells by delivering *Oct4* using the cell-permeable peptide, LMWP, which can enhance the in situ regenerative potential of somatic cells. MEF cells have stem cell-like properties via *Oct4*-dependent cellular reprogramming factors, especially when complexed with LMWP. The development of effective gene carriers requires many essential factors, including the efficient delivery capacity of target genes into the target cells, their stability in living systems, and their biocompatibility, which reduces toxicity [34,35,36]. Therefore, this study was designed to create a complex between *Oct4* and the non-cytotoxic LMWP to prevent the degradation of *Oct4* DNA (Figure 1, Figure 3 and Figure 4). Here, we revealed that the positively charged cell-permeable LMWP peptide helped condense the size of *Oct4* DNA complexes when in a 1:5 N:P ratio (Figure 2 and Figure 3). When the *Oct4*-LMWP complex was delivered into MEFs, stemness gene expression increased while fibroblast intrinsic properties decreased. These results suggest that endogenous cells can be converted through the derivation of stem cell transcription factors. 

In the field of regenerative medicine, direct conversion has advantages in situ and in the generation of clinically relevant cells, including hard-to-achieve cells, from easily obtained cells such as fibroblasts [37,38]. The viral carrier transport efficiency of gene delivery can be high, depending on the type of vector used [15,27]; however, the risk of integration of viral DNA into the host genome raises potential safety issues for potential therapeutics and poses concerns like inefficient/unpredictable reprogramming outcomes, genomic integration, and unwarranted immune responses and toxicity. Therefore, several non-viral delivery methods have been reported as alternative ways to incorporate the transcription factors necessary for direct cell fate conversion or to promote stem cell-like properties [39,40]. Emerging studies have reported LMWP fragments as possible nontoxic substitutes for protamine in clinical heparin neutralization [41]. These LMWP peptides, which displayed arginine-rich content in their composition, retained a nearly complete heparin-neutralizing ability but with significantly lower toxicity in vivo [35,42]. 

In this study, MEF cells treated with *Oct4*-LMWP successfully expressed mesenchymal stem cell-like properties, including cell surface markers and gene expression. As shown in Figure 7, the increased *Oct4* and *Nanog* expression levels in the *Oct4*-LMWP group indicated an increase in stem cell-like properties. The correlation between *Oct4* and *Nanog* is known to be proportionally related to the probability of cell differentiation and pluripotency [43], which suggests that *Oct4* DNA might affect *Nanog* expression levels, playing a role in maintaining pluripotency and in the reprogramming relationship between *Oct4* and Nanog [13]. 

Our analysis of fibroblast-specific gene expression in cells treated with the *Oct4*-LMWP complex showed differential responses to *Oct4* induction. According to the relationship between α-SMA and vimentin with stemness, *α-SMA* expression in somatic fibroblasts is correlated with stemness and proliferation markers [44]. In addition, previous studies have reported that vimentin, which is a biomarker of epithelial to mesenchymal transition, is required for cancer stem cell metastasis [45]. Mechanistic studies are still needed, but our results indicate that only the *Oct4*-LMWP complex changed fibroblast-specific characteristics after intracellular uptake of *Oct4* in MEFs. In addition, the *Oct4*-LMWP complex significantly increased the expression of stemness-specific markers without cell death.

Except for the viral carrier method, general protein-based direct conversion studies still have limitations. For example, the expression of mammalian-cell-derived proteins in *E. coli* often resulting in inclusion bodies [46]. In the process of denaturation and refolding in inclusion bodies, the recovery yield is reduced [47]. In addition, the inclusion bodies may re-aggregate due to misfolding during the refolding process or protein activity may be lost. Our results suggest that normal somatic cells can be converted into multipotent cells simply by using LMWP peptide as a target gene carrier. Moreover, we showed the effective intracellular uptake delivery of *Oct4* DNA by increasing the surface charge of the target gene through the complex formation with LMWP (Figure 4).

The stable delivery of recombinant reprogramming factors is still considered a major challenge for reprogramming strategies [48]. *Oct4* is a master regulator that needs to be maintained at high levels for effective reprogramming to induce stem cell-like properties, as well as for the direct conversion of cells into other lineages. In conclusion, the *Oct4*-LMWP complex is a relatively safe and efficient method of cellular reprogramming because it is intracellularly delivered and maintains transcriptional regulatory activity. Nevertheless, the human fibroblasts were not directly assessed in this study, and therefore, a further study on human fibroblasts with *Oct4*-LMWP would be necessary to validate the direct conversion for clinical therapy. The *Oct4*-LMWP complex could contribute to the development of feasible somatic cell reprogramming technology and thereby, contribute to enhanced regenerative efficacy. 

## 4. Materials and Methods 

### 4.1. Synthesis of Cell Permeable Peptides

Cell-permeable peptides, LMWP, were manufactured using a peptide synthesizer (Prelude, Protein Technologies Inc., Tucson, AZ, USA) based on standard 9-fluorenylmethoxycarbonyl (Fmoc) chemistry. Rink amide methylbenzhydrylamine (MBHA) resin (GL Biochem, Shanghai, China) was pre-swollen in dimethylformamide (DMF, 50 mg/mL) and the Fmoc-protecting groups of resin were removed with 20% piperidine in DMF. Amino acid coupling occurred in the presence of 10 equiv. DIPEA, 5 eq. HBTU, and 5 eq. Fmoc-protected amino acids according to the resin loading of amino acids. Cleavage and sidechain deprotection of the peptide resin was conducted for 4 h using a cleavage cocktail (trifluoroacetic acid: phenol: water: thioanisole: 1,2-ethanedithiol = 82.5:5:5:5:2.5). Solutions containing the cleaved peptides were precipitated by adding cold ether. Peptides were purified using preparative reverse-phase high-performance liquid chromatography (RP-HPLC; Shimadzu, Kyoto, Japan) with a Kromasil 100-10-C18 column (Nouryon, Bhous, Sweden) at 220 nm and a 40 min gradient from 95% to 5% water/acetonitrile containing 0.1% trifluoroacetic acid (TFA). Peptide purity was determined to be >98% by HPLC (Shimadzu, Kyoto, Japan) and liquid chromatography–mass spectrometry (LC-MS, Shimadzu, Kyoto, Japan).

### 4.2. Isolation and Culture of MEFs

Female pregnant C57BL/6J mice were supplied by Daehan Bio Link Inc. (Eumseong, Korea). All animal experiments were approved by the Chungbuk National University Institutional Animal Care and Use Committee (IACUC ID: CBNUA-1418-20-01). MEF cells were isolated from mouse embryos according to the guidelines. Briefly, embryos were acquired from pregnant mice at 13.5 d and washed three times with Dulbecco’s phosphate-buffered saline (DPBS; Welgene Inc., Gyeongsan, Korea). The embryos were separated from the uterine horn and the placenta. The isolated embryos were mechanically digested and then the tissues were chemically digested with DNase I (Sigma-Aldrich, St. Louis, MO, USA). The digested tissues were incubated with 0.25% trypsin-EDTA (Gibco, Grand Island, NY, USA) at 37 °C for 10 min. 

The mononuclear cells were seeded in high-glucose Dulbecco’s modified Eagle’s medium (DMEM-HG; Welgene Inc.) supplemented with 10% fetal bovine serum (FBS: certified, US origin, Gibco), 1% antibiotics/antimycotics (A/A), and penicillin-streptomycin (P/S; Gibco) and cultured at 37 °C in a humidified 5% CO_2_ incubator. Cells were allowed to adhere to culture plates for 12 h and adherent mononuclear cells were used as MEFs. When the MEF cultures reached 80–90% confluence, they were treated with 0.25% trypsin-EDTA (Gibco) for 3 min. The detached cells were washed twice with PBS and collected by centrifugation at 3000 rpm for 5 min (Eppendorf, Hamburg, Germany).

### 4.3. Preparation of Oct4-LMWP Complexes

The *Oct4*-LMWP complexes were formulated by adding varying ratios of LMWP (1:1, 1:2, 1:3, 1:5, 1:10 N:P ratio) to *Oct4* DNA (10 μg/mL). The complex was agitated at 37 °C for 1, 2, 3, 4, or 5 h. The optimal N:P ratio of LMWP for stable and effective complex formation with *Oct4* DNA was determined by gel retardation, serum stability, and DNase I stability assays. The *Oct4* DMA sequences of thiol oligonucleotide and 6-fluorescein amidite (6-FAM)-labeling were synthesized by Bioneer Technologies, Inc. (Daejeon, Korea) as follows: 5′-(Thiol)ATG GCA TAC TGT GGA CCT CAG GTT G(6-FAM)-3′. For the control Oct4-Lipo complex, transfection of *Oct4* DNA was carried out using Lipofectamine^TM^ 2000 (Invitrogen, Schwerte, Germany) according to the protocol of the manufacturer’s instructions. The day before transfection, 2.0 × 10^5^ MEFs were cultured in Opti-MEM^®^ (Invitrogen) and transfected with *Oct4* DNA. 

### 4.4. Measurement of Oct4-LMWP Complex Particle Size and Zeta Potential

The particle size and surface charge of each *Oct4*-LMWP complex sample formed at different N:P ratios and *Oct4*-Lipo were determined at 25 °C with a scattering angle of 90° using a Zetasizer (Malvern, UK). The *Oct4*-LMWP complexes were prepared in deionized water to ensure that their evaluation was conducted under low-ionic-strength conditions so that the surface charge of the particles could be accurately determined.

### 4.5. DNase I and Serum Stability of the Oct4-LMWP Complex

DNase I (50 units) was added to a solution of *Oct4* DNA alone and in the LMWP complex and mixed solutions were incubated for 1, 2, 4, or 6 h at 37 °C. During incubation, 50 μL of each suspension was collected at the different time points, mixed with 75 μL quenching solution (4 M ammonium acetate, 20 mM EDTA, and 2 mg/mL glycogen), and placed on ice. After incubation, each complex was subjected to 1% agarose gel electrophoresis. The stability of the *Oct4*-LMWP complex was investigated by incubating 100 μL of the complex in 100 μL 50% FBS solution for 1, 2, 4, or 6 h at room temperature. The incubated complex solutions were subjected to 1% agarose gel electrophoresis. Naked *Oct4* DNA was used as a control.

### 4.6. Cellular Uptake Assay 

To confirm the cell membrane permeability of the *Oct4*-LMWP complex, MEF cells were seeded (1.5 × 10^5^) onto a 6-well plate and incubated at 37 °C under 5% CO_2_. The FAM-*Oct4*-LMWP complex was added to the medium at each N:P ratio. Each group was fixed with 4% paraformaldehyde (Biosesang, Seongnam, Korea) and washed three times with PBS. Nuclei were counterstained with DAPI for 3 min. The cells were observed and imaged with a confocal microscope (LSM-880 with Airyscan, Oberkochen, ZEISS). For real-time live imaging of cellular uptake, cells were treated with DNA, lipofectamine, or *Oct4*-LMWP complex before live-cell imaging using laser scanning microscopy (Lionheart FX, Biotek, Korea). The cells were observed at 1 h intervals, maintained at 37 °C under 5% CO_2_ for 6 h.

### 4.7. Fluorescence-Activated Cell Sorting (FACS) Analysis 

The MEF cells (1.0 × 10^4^ cells) from the *Oct4*-Lipo and *Oct4*-LMWP (N:P = 1:5) groups were incubated with phycoerythrin (PE)-conjugated monoclonal antibodies against Isotype-PE and PE-conjugated CD34 (BD Biosciences, San Jose, CA, USA) for 30 min at 4 °C. Cell populations were analyzed using a FACScan instrument (FACSCalibur-S). Untreated MEF cells and isotype-PE Ig controls for each wavelength were used as controls. Data were analyzed using the FlowJo software (BD Biosciences).

### 4.8. Quantitative RT-PCR

To obtain RNA, MEF cells were seeded onto a 6-well plate and incubated at 37 °C under 5% CO_2_ until 70–80% confluence. Cells were incubated for 2 h in DMEM containing 0.5% serum for starvation. Total RNA from MEF cells was extracted using TRIzol reagent according to the manufacturer’s instructions (Life Technologies, Darmstadt, Germany). Isolated RNA was treated with DNase I (Thermo Scientific, Schwerte, Germany) to remove possible genomic DNA contamination and used for cDNA synthesis with Superscript III Transcriptase (Life Technologies) and oligo dT (Thermo Scientific) at 42 °C for 1 h. Each sample was run in triplicate. Quantitative RT-PCR was conducted using Luna Universal qPCR Master Mix (NEB, Massachusetts, USA) and 0.1 μM primers on the Magnetic Induction Cycler Real-Time PCR System (BMS, Coomera, Australia) under the following cycle conditions: primary denaturation at 95 °C for 5 min, 42 cycles of 30 s at 95 °C, 40 s at 60 °C, and 30 s at 72 °C, followed by fluorescence measurement. The primer sequences used were as follows: (1) *FSP1*: forward, 5′-GAT GAG CAA CTT GGA CAG CA-3′; reverse, 5′- ATG TGC GAA GAA GCC AGA GT-3′. (2) *Fibronectin*: forward, 5′-CCG TGG GAT GTT TGA GAC-3′; reverse, 5′- GGC AAA AGA AAG CAG AGG-3′. (3) *α-SMA*: forward, 5′-CTG ACA GAG GCA CCA CTG AA-3′; reverse, 5′-CAT CTC CAG AGT CCA GCA CA-3′. (4) *Vimentin*: forward, 5′-TCC AGA TCG ATG TGG ACG TT-3′; reverse, 5′-ATA CTG CTG GCG CAC ATC AC-3′. (5) *Oct4*: forward, 5′-CAG ACC ACC ATC TGT CGC TTC-3′; reverse, 5′-AGA CTC CAC CTC ACA CGG TTC TC-3′. (6) *Sox2*: forward, 5′-TGG CGA ACC ATC TCT GTG GT-3′; reverse, 5′-GGA AAG TTG GGA TCG AAC AAA AGC-3′. (7) *Nanog*: forward, 5′-GTC CCA AAG GCA AAC AAC CC-3′; reverse, 5′-GCT GGG TGG AAG AGA ACA CA-3′. (8) *β-actin*: forward, 5′-GAC AAC GGC TCC GGC ATG TG-3′; reverse, 5′-TGG CTG GGG TGT TGA AGG TC-3′.

### 4.9. Statistical Analysis

All data are presented as means ± standard deviation (S.D.) and were obtained from at least three independent sets of experiments to ensure the reproducibility of the results. The statistical significances of differences among each group (control, *Oct4*-Lipo, and *Oct4*-LMWP) were determined using one-way analysis of variance (ANOVA) followed by Tukey’s honestly significant difference test using GraphPad Prism 7 software (GraphPad Software Inc., La Jolla, CA, USA). Statistical significance between groups was analyzed using the Student’s *t*-test. The level of significance is represented as * *p* < 0.05, ** *p* < 0.01, or *** *p* < 0.001.

## Data Availability

The data presented in this study are available on request from the corresponding author.
